# Biomarkers in chronic adult hydrocephalus

**DOI:** 10.1186/1743-8454-3-11

**Published:** 2006-10-04

**Authors:** Andrew Tarnaris, Laurence D Watkins, Neil D Kitchen

**Affiliations:** 1 Victor Horsley Department of Neurosurgery, National Hospital for Neurology and Neurosurgery, Queen Square, London WC1N 3BG, UK

## Abstract

Awareness of the importance of chronic adult hydrocephalus has been raised again with the recent emergence of epidemiological studies. It is estimated that between 5 and 10% of patients suffering from dementia might, in fact, have chronic hydrocephalus. Although, surgical diversion of the cerebrospinal fluid (CSF) represents the only known procedure able to treat the symptoms of this condition, the selection of surgical patients has always been problematic. In the last 40 years, we have become wiser in using appropriate diagnostic tests for the selection of these patients; however, the area of biological markers has so far been overlooked in this condition, in contrast to that for other neurodegenerative disorders and dementias. Biomarkers are biological substances that may be used to indicate either the onset or the presence, and the progression of a clinical condition, being closely linked to its pathophysiology. In such a setting they might assist in the more appropriate selection of patients for shunt surgery. In this article, we have reviewed research carried out in the last 25 years regarding the identification of serum and CSF biomarkers for chronic hydrocephalus, discussed the potential for each one, and finally discussed the limitations for use, as well as future directions and possibilities in this field. It is concluded that tumour-necrosis factor, tau protein, lactate, sulfatide and neurofilament triple protein are the most promising CSF markers for chronic hydrocephalus. At present however, none of these meet the criteria required to justify a change clinical practice. In the future, collaborative multi-centre projects will be needed to obtain more substantial data that overcome the problems that arise from small individual and uncoordinated studies.

## Background

Chronic communicating hydrocephalus in adults is now recognised as one of the causes for reversible dementia, with an occurrence of 1–10% among patients with a diagnosis of dementia [1,2]. Data from Sweden suggest an incidence of 3.36 surgical operations for chronic hydrocephalus per 100,000 inhabitants per year [3,4]. However, it is thought that normal pressure hydrocephalus is significantly underestimated because many cases go unreported and untreated [5]. The term chronic hydrocephalus of adult-onset (CAH) encompasses a variety of conditions that include normal pressure hydrocephalus (NPH) of the idiopathic or secondary variety (depending on whether there is a known cause such as trauma, subarachnoid haemorrhage, meningitis, and tumour), communicating hydrocephalus due to a disturbance of CSF dynamics, non-tumoural aqueductal stenosis, and compensated arrested hydrocephalus. We are still in the process of understanding the pathophysiological mechanisms underlying hydrocephalus in adults, even though 40 years have passed since Hakim and Adams first coined the term "Normal Pressure Hydrocephalus" [6].

Biological markers have traditionally been used in clinical practice in order to support a diagnosis, or monitor the progression of a disease by measuring levels longitudinally. The definition as given by the Biomarkers Definition Working Group was "*A characteristic that is objectively measured and evaluated as an indicator of normal biological processes, pathogenic processes or pharmacological responses to a therapeutic intervention*."[7]. Biomarkers can assist us in this task as they provide an insight to the changes of the cerebral milieu associated with the condition. In order for their use to be established in routine clinical practice, they should demonstrate high sensitivity and specificity. Biomarkers have long been used in the neurosciences for these exact reasons [8-13]. Some well established biomarkers in the field of neurodegenerative disorders and dementias are the neurofilament light chain polypeptide for multiple sclerosis (MS) [14], and tau protein in Alzheimer's dementia (AD) [15]. Extensive research has already been carried out to document the hydrocephalus-induced changes in the composition of cerebrospinal fluid (CSF) [16].

Serum biomarkers have played an important role in other neurological conditions, due to the relative accessibility of samples [17,18]. However, CSF remains the primary fluid of choice to monitor these changes. The concept of the "*sink action*" of the CSF was first introduced by Davson in 1962 to highlight this potential [19]. The aim of this paper is to review the role that biomarkers play in the diagnosis and monitoring of the clinical progression of CAH, and to discuss current research and future perspectives. We have summarised the current literature in Table 1 [see Additional file 1].

### Search strategy

A Medline search using Pubmed and EMBASE databases was carried out for the years 1980–2005, using the key words *biomarkers, biological markers, CSF, serum, communicating hydrocephalus*, and *normal pressure hydrocephalus*. The search was limited to adults, and to abstracts in the English language. Thirty articles were retrieved and reviewed (see table 1). Articles from animal studies were reviewed and used only to support relevant human findings. A review of citations within the retrieved references was also carried out for earlier relevant references.

### Serum biomarkers

Despite serum being an easily accessible biological fluid, only four studies on biomarkers in adult hydrocephalus have been identified, thus demonstrating that there is an open field for the discovery of serum biomarkers in CAH. Vasopressin plasma levels were studied in 11 patients and compared to controls. No significant difference was found between the two groups [20] and similar results were found in another study with 18 patients [21].

Glycoprotein D2 serum levels were measured in 13 patients with NPH and there was no difference compared to control subjects. However, the mean values were significantly lower when compared to those of patients suffering from primary degenerative dementia of Alzheimer's type. Unfortunately, the authors do not comment on the significance of this finding and neither were the findings correlated with imaging or with CSF dynamic studies [21]. Preoperative melatonin levels were studied in six patients with NPH and were found to be lower than controls. Postoperative values did not differ significantly, but shunting restored the preoperative deranged diurnal rhythm of melatonin [22].

### Cerebrospinal fluid biomarkers

Biomarkers in the CSF are potentially more useful because they provide an insight into changes in the brain milieu associated with the condition, and consequently more research has been undertaken on the composition of the CSF. It is assumed that ventricular CSF will reflect the changes happening in the brain parenchyma, and more specifically in the periventricular white matter. Ideally, levels of biomarkers should be measured before and after surgical diversion of the CSF, in order to obtain clinically useful indices for the diagnosis and progression of the disease. Initial attempts took place in the early 80's [23]. There are some reasons why good experimental criteria have not always been met: 1) universally accepted outcome scales have not been produced until recently, making comparison between different studies and different groups difficult [24], 2) NPH is a relatively rare disorder and it is only recently that there is increased awareness about its importance, 3) CSF is not always available for repeated sampling, especially in pre-shunt patients with no accessible route into the ventricles (reservoir). However, this latter cohort of patients may provide a better insight into the pathophysiology, because after shunt insertion the CSF dynamics are altered.

### 1. Neuropeptides

#### • Somatostatin

Somatostatin (SOM) in the CSF has been measured by two groups, in 1991 [25] and in 2001 [26]. In the study by Wikkelso, CSF SOM levels were significantly lower (*p *< 0.05) in NPH patients when compared to the control group and Poca et al also observed the same. They suggested that the decrease could be the result of damage to the cortical neurons and the nerve terminals of the hypothalamus that normally have high concentrations of SOM. The normal concentrations of SOM in these structures are selectively impaired in experimental hydrocephalus [27,28].

Following shunting, SOM concentration significantly increased compared to preoperative values. The authors also detected a significant correlation with visual memory performance (*r *= 0.57; *p *= 0.032) and with visuomotor speed (*r *= -0.55; *p *= 0.05), demonstrating that higher concentrations of SOM were associated with better visual memory and increased speed of mental processing, features that are known to be deranged in NPH. These associations did not persist after surgery. After shunting, changes in SOM concentrations correlated significantly (*r *= 0.66; *p *= 0.01) with improved daily life activities (measured with the Rapid Disability Rating Scale-2 (RDRS-2). In another smaller study, levels of SOM were lower in the idiopathic NPH (iNPH) group when compared to controls, but were not correlated with either Mini Mental State Examination (MMSE) scores or the Blessed dementia scale [29]. The modulatory role of somatostatin in cognition has already been proposed [30].

#### • Vasoactive Intestinal Peptide

Wikkelsö in 1985 examined the role of the vasoactive intestinal peptide (VIP) in the pathogenesis of NPH and in multi-infarct dementias [31]. The preoperative concentration of VIP in CSF was significantly lower in NPH when compared to controls, but increased postoperatively. The rationale behind the study was that VIP is a potent vasodilator and therefore may play a role due to the presence of chronic ischemia [32]. These results were verified again in a later study [25] by the same group. Tullberg *et al*. compared the levels of VIP in 43 patients with NPH and 19 with subcortical arteriosclerotic encephalopathy (SAE) and found the CSF levels of VIP higher in patients with SAE than with NPH [33]. They also noticed that the group of NPH patients suffering from cerebrovascular disease demonstrated higher VIP concentrations than those with other aetiologies. They suggest that higher VIP concentration in patients with SAE could be due to activation of VIP-ergic neurons to accomplish a compensatory vasodilatation. This would not, however, explain why the VIP levels increased following a shunt operation as in the previous study, since cerebral blood flow has been shown to be restored postoperatively [34]. Tisell *et al *compared the levels of VIP in 18 patients with aqueductal stenosis and 19 patients with iNPH [35]. The authors correlated the results with outcomes, concluding that levels of VIP correlated weakly with postoperative deterioration in alertness.

#### • Delta-sleep-inducing peptide

Wikkelsö *et al*. investigated the role of delta-sleep-inducing peptide (DSIP) in the CSF of ten patients with NPH and compared the results with that of healthy volunteers and other dementias [25]. The levels of the peptides were again decreased in NPH compared to control levels, but increased significantly in parallel to the clinical improvement following shunting. Since, DSIP is a 9-amino acid peptide with a role in the normal sleep-wakefulness regulation, the results are not surprising, especially since the reduction was more pronounced in subjects with worse psychomotor performance.

#### • Neuropeptide Y

Reduced levels of neuropeptide Y (NPY) in patients with NPH has been found in several studies [25,26,36]. Furthermore, it appears that the levels increase following shunting [26]. Wikkelsö *et al*. investigated the role of this neuropeptide in ten patients with NPH compared to levels in other patients with dementia; he also examined and compared the levels longitudinally three months post shunting. The percent increase in concentration following shunting was strongly correlated with percent change in a functional/activity scale (RDRS-2) [25]. Tisell *et al *in a later study, compared the levels in 18 patients with aqueductal stenosis and 19 patients with iNPH [35]. The authors correlated the results with outcomes, concluding that levels of NPY correlated negatively (r < 0.40) with postoperative improvement in alertness. However, lower CSF levels when compared with controls have been found also in patients with AD [37], therefore reducing the specificity of this peptide and negating its potential role as a biomarker.

### 2. Neurotransmitters

Tullberg *et al*. extended the range of biomarkers in a later study by examining the role of 4-gamma aminobutyric acid (GABA) in addition to the two previously mentioned biomarkers [33]. Similarly to the previous study, they correlated the biomarkers with surgical results. No correlation was found with the results of shunt surgery and the CSF concentration of GABA. Malm *et al*. considering that a similar biochemical disturbance to that of patients with different forms of dementia, namely disturbance in the cholinergic, serotonergic and noradrenergic system would occur in patients with NPH, measured the levels of 3-methoxy-4-hydroxy-phenylglycol (MHPG), homovanillic acid (HVA), and 5-hydroxyindoleacetic acid (5-HIAA), acetylcholinesterase (AChE) and butyryl cholinesterase (BuChE) [38]. They did not observe any significant differences between patients with NPH, AD, multi-infarct dementia (MID), and controls regarding the concentrations of HVA, 5-HIAA and MHPG, BuChE. They noticed reduce AChE activity both in the NPH and AD group when compared to controls. The levels of these transmitters did not correlate with the degree of ventriculomegaly, dismissing the possibility of a dilutional effect. They observed a positive correlation between outflow conductance in the hydrocephalic group and the concentrations of HVA, and 5-HIAA. Acetylcholinesterase activity was positively correlated with MMSE scores. The study highlighted the possibility of a common cholinergic disturbance in NPH and AD.

Homovanillic acid was further studied by Hildebrand *et al *as a reflection of dopamine metabolism, attempting to correlate the concentration with the clinical triad of gait disturbance, dementia and urinary incontinence [39]. No correlation was found in their study and the authors rejected the potential of HVA as a biomarker for this condition. Similarly, levels of HVA did not differ significantly between patients with NPH and controls in another study [33]. Another study did not find any difference in the levels of MHPG, HVA and 5-HIAA between cases of obstructive hydrocephalus and iNPH [40].

### 3. Cerebral metabolites

#### • Lactate

Lactate, an end product of anaerobic glycolysis, possibly represents the element of chronic ischemia implicated in the pathophysiology of normal pressure hydrocephalus. Lactate was studied by Malm *et al*. in 15 patients with iNPH and was significantly reduced when compared to controls (n = 21) [38]. Furthermore, they noticed a positive correlation between outflow conductance in the hydrocephalic group and the concentrations of lactic acid. One would expect the levels of lactate to be increased on a background of chronic ischemia. Malm explained this paradox by hypothesizing that a) there is an autoregulatory clearance of lactate out of the CSF via a facilitated transport mechanism in the choroid plexus and the cerebral subarachnoid space, b) due to inverse, caudo-rostral, flow of CSF, there is an accumulation of the metabolite in the ventricles and consequent transependymal absorption of lactate, and c) as a result of atherosclerosis and the pathological changes induced there is an obstacle to transependymal diffusion of lactate from brain extracellular fluid to CSF. Nooijen *et al*. however, reported higher lactate levels in the NPH group when compared to controls, and significantly higher values when compared with AD (*p *= 0.0005, n = 36) and vascular dementia (*p *<= 0.01, n = 49) concluding that lactate levels might differentiate between adult hydrocephalus and patients with Alzheimer's and vascular dementia [41]. Although the latter is a large cohort observational study with 57 hydrocephalic patients, the authors did not report correlation with CSF outflow conductance, ventriculomegaly or with surgical outcomes.

### • Free radicals

Fersten *et al*. studied the role of free-radical peroxidation products in the CSF of 24 patients with NPH, and in particular that of thiobarbituric acid-reactive material (TBAR), and protein sulfydryl (SH) groups [42]. The rationale behind the study was that free-radical peroxidation alters the structure of biological membranes and may therefore be implicated in the pathogenesis of chronic adult hydrocephalus. The results showed a significant increase in the levels of TBAR, total and soluble SH groups, as well as a decrease in the number of protein thiol groups between the NPH and the control group. The authors imply that peroxidation, which damages the cytoplasmic membranes, might be one of the factors that affect cognitive functioning.

### 4. Enzymes

#### • Neuron Specific Enolase

Nooijen *et al*. studied the CSF levels of neuron specific enolase (NSE), a glycolytic enzyme localized in neurons, in 57 patients with NPH. The levels were lower than both the control and the Alzheimer's group. However, this difference was not statistically significant and certainly was not correlated with surgical outcomes [41]. NSE, was significantly higher in the Alzheimer's and vascular dementia group as compared to the control group, while it did not differ significantly between the two dementia groups [43]. However, these results were contradicted in another study [44]. These findings are only suggestive that CSF-NSE has potential as a non-disease specific marker for the neuronal degeneration in dementia. NSE as a biomarker in hydrocephalus remains even less well established.

#### • Plasminogen Activator Inhibitor-1

Sutton *et al*. measured the levels of the plasminogen activator inhibitor-1 (PAI-1) in the CSF of patients suffering from various neurological disorders [45]. In the hydrocephalic patients the levels were not increased compared to the levels of control subjects. Interestingly enough, the levels were increased in the case of Alzheimer's-type dementia, cerebral infarction and CNS infection. This finding contradicts results from newborns where high levels have been found in cases of posthaemorrhagic hydrocephalus [46,47]. PAI-1 is a 52-kDa glycoprotein, which under normal conditions is relatively restricted from entry into the CSF. The study found increased levels particularly in patients with brain tumours, pointing to possible alterations in the blood-brain barrier. The small number of patients in the above study, the negative findings, and the fact that the PAI-1 is found in a variety of neurological diseases excludes the use of this protein as a potential biomarker for hydrocephalus. However, PAI-1 may play a role in fibrinolysis occurring after a haemorrhage within the CNS. In the absence of adequate fibrinolysis, micro thrombi will obstruct the arachnoid villi and subsequently cause fibrosing arachnoiditis affecting the CSF dynamics.

#### • Prostaglandin D synthase

Mase *et al*. measured the levels of prostaglandin D synthase (PGDS) in 14 patients with normal pressure hydrocephalus and found them significantly lower when compared with a control patient and other patients with dementia patients (Lewy body dementia, vascular dementia, Alzheimer's type) [48]. This enzyme is produced in the leptomeninges, and the trabecular cells of the arachnoid membrane and then secreted in the CSF as beta-trace. The authors conclude that the observed decrease is probably due to a degenerative change in the arachnoid membrane and cannot be considered the cause of neurological symptoms in the case of NPH. It is unclear whether these decreased levels were due to decreased CSF production that reflect arachnoid damage, mere dilution, or to the disturbance in fluid dynamics.

### 5. Neural cell-derived proteins

#### • Myelin Basic Protein

Myelin Basic Protein (MBP) is a known indicator of brain damage and in particular of demyelination [8,49]. It is known that in hydrocephalus there is demyelination of the periventricular white matter and so MBP appears as an attractive marker to study the degree of this pathological process. Sutton *et al*. measured the levels of this protein in the CSF of hydrocephalic patients with different aetiologies and proposed that active hydrocephalus produces significant periventricular demyelination, probably as the result of mechanical stretching [34]. Interestingly, the degree of ventriculomegaly was positively correlated with the levels of MBP. The findings become more interesting since we know from the studies of Whitaker *et al*., that cerebral atrophy is not associated with elevated MBP values [50]. Later, Longatti *et al*. in 1993 examined the levels of MBP pre- and postoperatively. In their study of 17 patients with hydrocephalus who underwent surgical CSF diversion they observed that the levels of MBP decreased following the shunt operation, suggesting that MBP is an index of brain damage and its levels could be used as an indication for shunting [51]. They have not however correlated the levels with shunting outcomes. However, high levels of MBP before shunting may be explained by the pooling of molecules in stagnant CSF, which then decrease after flow is restored by shunting. In another study of 57 patients with NPH, the levels of MBP did not differ significantly between patients with NPH, vascular dementia, AD, and controls [41]. However, the levels of MBP were higher than controls.

Although studies have shown that the MBP levels decrease in humans postoperatively, different results were obtained in rats. In order to examine the possibility that neurons and oligodendrocytes, both of which represent deteriorating cell populations in hydrocephalus, can be regenerated by proliferating brain cells, rats with kaolin-induced experimental hydrocephalus were later injected with bromodeoxyuridine (BrdU). The BrdU positive cells for MBP were increased from 17% in the hydrocephalic group to 33% at an early stage after the shunt procedure, but were restored to 6% at a later stage after shunting. The differentiation to mature oligodendrocytes appears to be inhibited in hydrocephalus even after the shunt procedure [52]. Del Bigio *et al*., who measured the degree of myelination indirectly by measuring the MBP in cerebrum of rats with experimentally induced hydrocephalus, observed that with persistent hydrocephalus, the corpus callosum became thinned, axons were lost, and myelin-related enzyme activities and proteins were decreased. The timing of intervention became important as he showed treatment of hydrocephalus at 1 week largely prevented the damage while shunting at 4 weeks failed to restore the injured white matter concluding that hydrocephalus in the immature rat brain delays myelination, but compensatory myelination is possible if treatment is instituted prior to the development of axonal injury [53]. This finding correlates with our clinical experience on NPH and the importance of shunting patients as soon as possible in order to achieve the best clinical outcome.

#### • S-100b

The presence of this protein in blood points to the functional and/or morphological disruption of the blood-brain barrier [54]. It is a major protein of the cytosol predominantly found in glial cells. Increased levels have already been found in cases of astrogliosis [55] and hydrocephalic children [54]. S-100b levels in the CSF, were studied by Nooijen *et al*. who showed that S-100b levels did not differ between patients with NPH (n = 44), and controls [41], and therefore its role as a marker in chronic adult hydrocephalus is doubtful.

#### • Nerve Growth Factor

Nerve Growth Factor (NGF) is known to promote neuronal recovery from injury and age-related atrophy, being also important in the regeneration in the brain. NGF is not normally detectable in innervated tissues, but ablation of the innervating neurons leads to the production of measurable NGF in the target tissues [56]. Increased NGF mRNA levels have been detected in the medial septal nucleus, striatum and corpus callosum in experimentally-induced hydrocephalus in rats [57]. Yang *et al*. investigated the role of NGF in the pathogenesis of hydrocephalus. They measured the levels of NGF in nine adult patients with high pressure hydrocephalus (the authors define high pressure as CSF pressure >10 cm H_2_0) and seven patients with ex-vacuo hydrocephalus [58]. The levels were significantly higher postoperatively in the second group, despite the fact that no significant difference existed perioperatively. The results suggested that the neuronal injury was more severe in the ex-vacuo category, however one may argue that the last measurement was made only four days postoperatively. No correlation was made with surgical results. In a similar study Hochhaus *et al*. measured the levels of NGF and neurotrophin-3 (NT-3) in 42 hydrocephalic children. The levels of both were again elevated in comparison to controls, however the results were only correlated with presenting symptoms and not with outcomes [59]. Increased levels of NGF when compared to controls have been verified in another study of 16 children with communicating hydrocephalus [56]. These findings suggest the possibility that the elevation of NGF concentration in CSF was caused by increased generation of glial cells that resulted from brain damage.

#### • Tau protein

Kudo *et al*. studied the role of tau protein by measuring the levels in 20 patients with NPH. Tau concentrations were elevated compared to those of orthopaedic controls [60]. The results of his study were not correlated with surgical outcomes. The levels of the protein were positively correlated, however, with the severity of dementia and with urinary incontinence but not with gait. Tisell *et al *also compared the levels of tau protein in 18 patients with aqueductal stenosis and 19 patients with iNPH, concluding that the levels of tau have no correlation with clinical improvement [35]. Tau protein, a microtubule-associated protein has been found to be elevated in the CSF of patients suffering from Alzheimer's disease [15], as well as in patients with Lewy body dementia, corticobasal degeneration [61], and Creutzfeldt-Jakob disease [62] indicating that it is a marker of neuronal degeneration.

Lins *et al*. measured the immunoreactivity of amyloid beta peptide (1–42, Ab42-IR) and tau protein (total tau immunoreactivity (TTIR) in 12 patients with NPH, and compared them with the levels of an equal number of patients suffering from vascular dementia, Alzheimer's disease, Parkinson's disease without dementia and 24 controls [63]. TTIR levels in NPH were not significantly changed when compared with the other causes of dementia and controls, whereas Ab42-IR was significantly decreased when compared with Parkinsonian patients and control subjects. The authors combined the results of both markers in a single plot as a method to discriminate between different groups of dementia; all the NPH patients were within the predicted area. Increased levels of TTIR are believed to reflect ongoing neuronal and axonal degeneration or damage, whereas decreased Ab42 may be the result of increased recruitment of Ab42 from the CSF and the brain interstitial fluid to deposits in the form of plaques or decreased secretion into the CSF [64]. A recent experimental study in rats with kaolin-induced hydrocephalus verified the above results showcasing increased accumulation of Ab42 in the periventricular area, around the cortical vessels and the cortical parenchyma; the size of the deposits correlated well with the duration of the condition [65].

#### • Glial Fibrillary Acidic Protein

GFAP is a non specific marker indicating gliosis [66,67]. Tullberg *et al*. were the first to find a useful biomarker associated with the progression of NPH. They measured the CSF levels of GFAP in 65 patients with NPH (21 of which had iNPH), and correlated them with preoperative clinical presentation and signs. They observed a two-fold increase in GFAP levels when compared to controls [68]. Similar results regarding GFAP were verified in an earlier study by Albrechtsen *et al*. [69]. GFAP did not seem to correlate with severity of symptoms or presentation and had no correlation with the outcome of shunt surgery. In particular, GFAP in CSF suggests an irreversible damage to astrocytes, since GFAP is not secreted by astrocytes.

#### • Neurofilament triplet proteins

Neurofilament proteins are Type III intermediate filament proteins that assemble into neurofilaments, the major cytoskeletal element in nerve axons and dendrites. They consist of three distinct polypeptides, the neurofilament triplet protein (NFL). It has been shown that the metabolism of neurofilaments is disturbed in Alzheimer's disease [70]. Therefore, NFL may be used as a biochemical marker of neuronal degeneration and particularly of axonal damage [67]. Tullberg *et al*. measured the levels of NFL in 65 patients with normal pressure hydrocephalus (21 of the idiopathic type) and correlated them with preoperative clinical presentation and signs. They observed a six-fold increase in NFL levels when compared to controls. NFL as the authors point out is not specific for NPH and therefore cannot differentiate between various types of dementia. This increase in NFL indicates a degeneration of neurons primarily affecting the axonal region with a loss of intermediate filament protein across deranged cell membranes into the interstitial fluid. Although, they report outcome results without using any of the commonly used scales, in the case of NPH they conclude that high preoperative NFL levels are associated with favourable surgical outcomes (*r *= 0.3, *p *<= 0.05), and suggest that NFL can be used as a marker for ongoing axonal damage [68]. However, in another study from the same group, even though the increased levels of NFL in the ventricular CSF of patients with iNPH were verified, the results did not correlate with improvement [35].

#### • Sulfatide

Sulfatide is a glycosphingolipid component of myelin and it has been recently understood by experiments in sulfatide-null mice, to be essential for the maintenance of CNS myelin and axon structure [71]. In an early study, the role of sulfatide was studied in patients with communicating hydrocephalus [33]. The preoperative CSF sulfatide levels were found to be higher in the NPH group with cerebrovascular aetiology, when compared with the rest of the NPH patients. The authors postulated the presence of irreversible ischemic white matter lesions in the hydrocephalic group with cerebrovascular disease. Since the sulfatide levels were normal in most of the NPH patients, they considered that demyelination plays a minor role in the pathogenesis of NPH. In addition, there was no correlation with surgical outcome. The authors state that the results cannot be explained by a difference in CSF dynamics and therefore the concentration of sulfatide can differentiate between NPH and subcortical arteriosclerotic encephalopathy (SAE). The latter condition also known as Binswanger's disease has a similar clinical presentation to NPH [72], and therefore this contribution is significant in establishing the diagnostic role of sulfatide as a biomarker. Tisell *et al *compared the levels of sulfatide in 37 patients with aqueductal stenosis and iNPH [35]. The levels of sulfatide correlated inversely with improvement in psychometric performance; this correlation however was weak.

#### • Glycoprotein D2

Glycoprotein D2 is a glycoprotein enriched in neuronal membranes and probably involved in intercellular adhesion. The levels of this protein were examined in the CSF of 13 hydrocephalic patients and compared with controls and patients suffering from primary degenerative dementia of Alzheimer's type. The levels were significantly lower in the case of NPH. No correlation was made with symptoms, surgical outcomes or outflow conductance. The significance of this study remains unknown in the setting of NPH [21].

### 6. Cytokines

Tarkowski *et al*. investigated the role of the tumour-necrosis factor (TNF-α), an inflammatory mediator, and whether NPH triggers its production [73]. They examined the levels of TNF-α in the CSF of 35 patients with NPH and compared them with controls. In the NPH group the levels were 45-fold higher. The most interesting finding was that TNF-α returned to control levels following shunting in the group that improved following surgery. This factor has a short half-life in CSF, hence accumulation due to CSF stagnation is unlikely, and the increase may be due to increased production preoperatively. The authors also suggest that TNF-α might be a marker for demyelination and suggest TNF-α toxicity is directed to the white matter in patients with NPH.

Recently, the CSF levels of two interleukins, IL-4 and IL-10, have been compared in different neurodegenerative diseases [74]. Levels of both interleukins were significantly higher when compared to controls, but not different when compared to patients with dementia and CJD. The authors suggest that elevated levels of those cytokines might reflect a response to neurodegeneration, and also might trigger neuroregeneration. In another study IL-1 levels in the CSF of patients with AD were significantly higher when compared with NPH [75]. The results of both studies were not correlated with surgical outcomes. This, together with the small number of patients, means that it is not possible to obtain any solid conclusions.

### Other biomarkers

We will briefly summarise below studies of biomarkers that have been investigated less, or only in single studies. CSF levels of vasopressin did not differ between NPH patients and controls in two studies of 11 and 18 patients, respectively. Hammer *et al*. suggested that there might be a role of vasopressin affecting the memory, however the results of the study did not substantiate this claim [20]. The concentrations of corticotropin releasing factor (CRF) were examined in 14 patients with NPH pre- and postoperatively [26]. The levels of CRF increased significantly post-shunting, nevertheless they remained below normal levels. The authors explain this difference by a possible improvement in cerebral blood flow that is known to occur post shunting. They also noted that the change in CRF correlated negatively with percentage change in postoperative verbal fluency and in the trail-making test B, a test measuring psychomotor speed. No other study has been identified in the literature and unfortunately we cannot gain a better insight into the role of CRF with hydrocephalus. Levels of cholecystokinin, a 33-amino acid polypeptide acting as a neurotransmitter or neuromodulator [76], were found to be significantly lower in 16 patients with NPH when compared with controls and also lower levels were correlated with abnormal intracranial pressure (ICP) values [77]. Brettschneider *et al*. studied the CSF levels of leptomeningeal derived β trace protein, beta2 microglobulin and cystatin C in groups of patients suffering from NPH (n = 19), AD (n = 30), vascular (n = 13) and frontotemporal dementia (n = 6). The levels of β-trace protein were lower in the NPH group than the controls and patients with AD suggesting a meningeal dysfunction in the pathogenesis of NPH. The authors suggest this protein is a potential biomarker discriminating between NPH and AD, although no correlation was carried out with surgical outcomes [78].

## Discussion

During the recent years, various groups have made efforts to identify CSF markers for neurodegenerative disorders. These efforts have so far been concentrated on other neurogenerative disorders such as Alzheimer's dementia [79], Parkinson's disease [80], Pick's disease, and Lewy Body dementia [81]. Although improvements of clinical NPH symptomatology have been described after shunting in patients with neuropathologically confirmed concomitant Alzheimer's dementia [82], it has been shown that NPH patients with additional pathology attributed either to vascular dementia or Alzheimer's disease generally show worse outcomes after shunting than those with NPH without concomitant pathology [83]. The pathology of these conditions is so closely interlinked [53,84,85] that Silverberg *et al*. even suggested a low-flow (up to 140 mL/d) CSF drainage pilot study for patients with Alzheimer's dementia [86]. Therefore, neurochemical parameters that could help to separate NPH from other neurological disorders, which mimic NPH symptomatology, would be of clinical value. Pathological studies have given us an insight into the changes occurring in chronic hydrocephalus. Disruption of the ependymal ventricular lining, interstitial oedema, neuronal degeneration, white matter lesions, gliosis, capillary micro-infarctions and demyelination [45,87-90] are consistent findings. Studies from experimentally-induced hydrocephalus in rats have supported these findings and provided additional evidence [91,92]. Furthermore, we now know that a regenerative process takes place following gradual necrosis in the white matter and axonal injury, and as a response there is the production of new glial cells in the subependymal zone to compensate for the cell loss [93].

The case for identifying biomarkers in chronic hydrocephalus of adult onset has arisen due to similar developments in other common causes of dementia and the increasing awareness of both the epidemiology of NPH [3,5] and its impact on the quality of life of elderly patients [94]. Ideally, useful biomarkers should be confirmed with data from multiple disciplines, including neuropsychological testing, blood tests, genetic markers, CSF composition, and brain imaging. Alterations in the neurochemical composition of CSF in hydrocephalus have been widely documented and reviewed [16]. Newer techniques, which will be discussed in the next section, will provide us with a broad spectrum of biological markers ranging from serum proteins to intracellular mediators that are involved in signal transduction and transcription [95].

### Definition of a biomarker and applications in patients with chronic adult hydrocephalus

"*The ideal biomarker for Alzheimer's disease (AD) should detect a fundamental feature of neuropathology and be validated in neuropathologically-confirmed cases; it should have a sensitivity of 80% for detecting AD and a specificity of 80% for distinguishing other dementias; it should be reliable, reproducible, non-invasive, simple to perform, and inexpensive. Recommended steps to establish a biomarker include confirmation by at least two independent studies conducted by qualified investigators with the results published in peer-reviewed journals*." This definition given by the Consensus Report of the Working Group on Molecular and Biochemical Markers of Alzheimer's disease (Ronald and Nancy Reagan Research Institute and the NIA Working Group-1998) adequately highlights the problems that may be faced in attempts to establish biomarkers for adult-onset communicating hydrocephalus. The role of a biomarker is to confirm a diagnosis, serve for epidemiological studies, assess for prediction, monitoring the progression and response to treatment and studying brain-behavior relationships. Any marker will need to be validated against a definite diagnosis. Traditionally the diagnosis of NPH was given only postoperatively on the basis of improvement of the patient. Recently a collaborative attempt was made to categorize patients with NPH as 'possible', 'probable' or 'definite' [96]. If this categorization were to be used, then biomarkers should only be tested in probable cases to achieve high diagnostic accuracy. The establishment of control groups is another problem that needs a solution. Ideally, the spouses of hydrocephalic patients could be used as controls, as they would be well matched for age, and environmental and lifestyle factors. However, medical ethics prohibit us from using invasive procedures (such as serum or CSF sampling) for research purposes in healthy individuals. Since one single biomarker might be inadequate to provide the needed diagnostic accuracy, a combination of more than one biomarker might give a solution. An example of this was the study by Lins *et al*. as analyzed in an earlier section of this report [63], and as has already been demonstrated in other neurodegenerative processes [97].

### Data synthesis

As we have seen, serum biomarkers appear to be no use at the present time because relevant studies have been so few. Serum Glycoprotein D2 was inadequately studied and therefore may not be used in the differential diagnosis with patients suffering from primary progressive dementia. Clearly, serum protein concentrations can be influenced by many factors other than brain and CSF composition, therefore a direct reflection of brain metabolism is likely to prove more useful. Studies of biomarkers in the CSF have been much more numerous although, as our review has shown, no biomarker has received enough attention from researchers to emerge with the needed specificity and sensitivity.

In most of the studies so far there is an observed difference in the levels of the biomarker preoperatively and postoperatively, but there are weak correlations with surgical outcomes. In other studies there are observed differences between patients and a control group, but rarely are results correlated with surgical outcomes. TNF-α emerges as a potential biomarker. Although the results were not correlated with surgical outcome, the 45-fold preoperative increase and return to normal post- shunting, shows the potential of this marker. However, only one study of 35 patients, reported results for TNf-α, and therefore the data has yet to be replicated by other research groups. Somatostatin appears to relate to changes and general impairment of cognitive functions, but bigger studies are warranted to highlight better this relationship. A significant correlation was also found between levels of tau protein in patients with NPH and the severity of dementia; again these results need to be replicated. Sulfatide emerged as a promising biomarker showcasing a sensitivity of 74% and specificity of 94% in distinguishing between NPH and subcortical arteriosclerotic encephalopathy. Also, levels of neurofilament triplet protein >640 mg/L have been identified by Tullberg *et al*. as having a predictive value of 100% for a positive outcome after shunt surgery; the latter remain non-sensitive (17%) but highly specific (100%). Lactate appears a promising distinguishing factor between different forms of dementia and further studies correlating lactate levels with surgical outcomes could reveal its potential as a biomarker.

### Technical considerations and existing limitations

There are a few problems of technical nature with using establishing and validating the use of biomarkers in this condition. These have been reviewed and acknowledged by Wood [98]. Firstly, caution should be exercised when interpreting biochemical results as for many potential markers there might be no reference values in the CSF of healthy subjects. There is also a need for age-matched reference values in the evaluation of CNS pathologies [99]. Wikkelsö *et al *have noted that in hydrocephalic patients, low neuropeptide values in the CSF can be caused not only by reduced release or increased degradation, but also by an altered distribution volume of the CSF [25]. Indeed this effect was noted with the MBP studies, levels of which decreased after flow was restored by shunting. Furthermore increased CSF levels of certain markers might be explained when one takes in consideration the low CSF conductance (i.e. increased CSF outflow resistance) in NPH which compromises the clearance of these metabolites resulting in accumulated levels in the CSF. Another controversial aspect of studies on concentrations of neuropeptides using lumbar instead of ventricular samples is whether the effect of the gravity may influence the concentration of the peptides in the samples [100,101].

The dynamics of markers in the CSF differ depending on whether the proteins derive directly from the nerve cells, the leptomeninges, or whether they derive from blood and therefore their concentration and dynamics depend on the integrity of the blood-brain barrier [102].

Ideally only ventricular samples would be used in the case of hydrocephalus, even though the spinal absorption pathways have been shown to be involved in the case of communicating hydrocephalus [103]. That is because the ventricular fluid would reflect more accurately the changes occurring in the periventricular white matter. Ideally the technique of microdialysis, which has been developed to quantify regional spatial and temporal changes in brain biochemistry, would be the best tool to monitor such changes. Indeed, there are publicized attempts in applying this in patients with NPH [104,105]. However, ethical issues and technical limitations for detecting putative markers might be the key issue in pursuing this further. It has also been demonstrated that concentrations of certain markers in the CSF might fluctuate over time, so a sample at one time point might be of limited use [106]. The sampling during ventricular catheterization seems to influence the concentrations of certain markers as verified by two studies [107,108]. This potential pitfall should be kept in mind in the design of relevant future studies. The differences in the pathogenesis of the idiopathic and the secondary form of chronic adult-onset hydrocephalus add more limitations for data collection from appropriate populations.

### Future directions and possibilities

Proteomics, since its inception in 1995 [109] has showed great promise in providing a more detailed insight into the mechanisms of disease in the post-genomic era. The proteome and peptidome maps can provide us with what is called "a bird's eye view" of the physiological and pathological products and of processes occurring at any one time. The field of clinical proteomics is especially well suited for discovery of biological markers in complex biological fluids, such as plasma, urine, serum and CSF; these in turn reflect the ongoing biological processes in healthy subjects, as well as in several neurodegenerative disorders. The related field of peptidomics is a method for analysing the vast range of the peptides that have been expressed in any cell, tissue or fluid at any given time. These two technologies have already been used successfully to provide us with an insight into the CSF proteome map [110-114].

Changes in the protein composition of CSF may be indicative of altered CNS protein expression pattern, providing us with a link to the cause or diagnosis of a condition. Other areas of neurology have already benefited from this new area. Mass spectrometry has been used in immunodeficiency virus dementia [115], in CSF from patients with multiple sclerosis [116], and Alzheimer's disease [117], or for the identification of protein tumour markers in primary brain tumours [118].

As with every new diagnostic technology, limitations do exist. At present there is a lack of standardization for the procedures as the techniques are quite new. A low total-protein concentration, a high concentration of albumin and immunoglobins, and a wide range of protein concentrations cause several difficulties in the identification of low-abundance CSF proteins [114]. An attempt to apply proteomics has already been carried out in the H-Tx rat inherited model of hydrocephalus [119]. A recent attempt in one single patient identified 82 proteins of which 25 have not appeared in any previously published two-dimensional electrophoresis (2DE) map of CSF, whereas eleven of them have not been previously reported to exist in CSF [111]. Two new peptides related to neuropeptide FF, a modulator of the opioid system, were detected in the CSF of one patient with NPH [120]. The significance remains unknown at present, but these two studies demonstrate the potential to gain a novel insight into areas that hitherto have not been available.

### The need for future collaboration

There is a need for continued and collaborative collection of research populations of subjects with chronic communicating hydrocephalus who can contribute to a longitudinal bank of biologic specimens (i.e., imaging data, biological fluid and genetic samples) for identification and verification of novel biomarkers. Similar projects in neurosurgery regarding brain injury (BrainIT) have been recently attempted and are ongoing. However, since NPH is mostly a clinical diagnosis with a radiological verification, such a biomarker might not be forthcoming even by the combined efforts of a controlled multicenter trial. Moreover, collaboration is needed between groups, which deal with paediatric and adult-type of hydrocephalus. Although, limitations exist due to the need for data protection, the long-term gain into the insight in the continuum spectrum of this condition will be immense.

Since follow-up and knowledge of the long-term prognosis for the management of this condition is vital, it is necessary to establish common outcome scales which will allow for multiple study comparisons. As we have seen outcomes and end points vary widely between groups dealing with this condition. This effort might also include establishing a national brain bank project for future collaborative research.

Attention should be also given in understanding the epidemiology of this condition.

So far, epidemiological data arises mainly from Sweden due its health system infrastructure. This approach could also include multiple non-medical disciplines that ultimately influence the outcome of geriatric conditions.

## Conclusion

From our literature search, TNF-α, tau, lactate, sulfatide and neurofilament triple protein in CSF have emerged as the most promising markers of chronic hydrocephalus. However, very few studies to date have reported results of putative biomarkers that have changed our clinical practice or the way we diagnose chronic adult-type hydrocephalus. Most importantly, no single biomarker has fulfilled all the criteria required. There is currently limited evidence supporting the role of biomarkers in chronic adult hydrocephalus, but it is not sufficient to support a change in clinical practice. Contributing factors to this, despite 25 years of research, is the fact that the results from different groups with different philosophy of management have been inconsistent with criteria regarding the diagnosis or management of this condition. Furthermore, the small number of patients in these studies prohibits us from presenting results with adequate statistical power that may influence or change our current practice. As with other areas of research these problems may be surpassed by collaborative multi-centre projects. Newer bioinformatic technologies are emerging as promising methods to successfully identify biomarkers.

## Competing interests

The author(s) declare that they have no competing interests.

## Authors' contributions

AT conceived of the review, carried out the literature search, and wrote most of the paper. LDW commented and proofread the paper. NK commented on the final draft. All authors have read and approved of the final version of this paper.

## Supplementary Material

Additional File 1 Review of literature. The table presents the review of the above literature and major findings in chronological order. Click here for file
