# Supplementary material for: Biomarkers in chronic adult hydrocephalus
Source: Cerebrospinal Fluid Res. 2006 Oct 4;3:11. doi: 10.1186/1743-8454-3-11 (PMC1617118; doi:10.1186/1743-8454-3-11)
Supplement: Additional File 1 — Review of literature. The table presents the review of the above literature and major findings in chronological order. [file 1743-8454-3-11-S1.doc]

**Table 1. Review of literature**

* Only abstract read from articles marked with asterisk.

| Author [Refe-rence] | Year | Hydroce-phalus type | Number of patients with CAH examined | Controls | Sample type | Biomarker | Findings | Conclusions |
| --- | --- | --- | --- | --- | --- | --- | --- | --- |
| **Hammer [1]** | 1982 | NPH (and BIH) | 11 | YES | Serum and CSF (lumbar and ventricular) | Vasopressin | AVP CSF levels were not different from the control group. AVP levels from LP reflected ventricular AVP levels. CSF levels were lower than plasma levels | CSF AVP levels in NPH were not different to controls, despite the degree of dementia in the hydrocephalic group. |
| **Sutton [2]** | 1983 | NPH (and other forms of hydrocephalus) | 5 | NO | CSF (lumbar and ventricular) | Myelin Basic Protein | MBP increased in 80% of NPH patients | Findings suggestive of periventricular demyelination |
| **Sorensen [3]** | 1983 | NPH (and primary degenerative dementia of Alzheimer’s type) | 18 | YES | Serum and CSF | Vasopressin | No difference in CSF and plasma vasopressin levels when compared with controls. |  |
| **Sorensen [4]** | 1983 | NPH (and primary degenerative dementia of Alzheimer’s type) | 13 | YES | Serum and CSF | Glycopro- tein D2 | Lower levels in NPH when compared with other groups. No correlation b/w levels and outflow conductance | Might be of diagnostic value in discrimination b/w NPH and PDD |
| [**Ahlberg**](http://www.ncbi.nlm.nih.gov/entrez/query.fcgi?db=pubmed&cmd=Search&itool=pubmed_Abstract&term="Ahlberg+J"%5BAuthor%5D)  **[5]** | 1985 | NPH (and other dementias) | 16 | NO | CSF (lumbar) | Adenylate Kinase | No difference in the levels in patients with NPH, multi infarct dementia and Alzheimer’s disease. | Although a marker of cerebral ischemia no increase was observed. |
| **Albrecht-sen [6]** | 1985 | NPH | 12 | YES | CSF (lumbar and ventricular) | GFAP | ↑ levels when compared to controls. | GFAP may be an aid in discriminating between NPH and patients with ventriculomegaly associated with neurodegenerative diseases. |
| **Wikkelso [7]** | 1985 | NPH (and other dementias) | 18 | YES | CSF (lumbar) | VIP | VIP levels increased significantly in patients with hydrocephalus 3 months post shunting. | There was a correlation of VIP levels with surgical outcome |
| **Kanki * [8]** | 1988 | NPH |  | YES | CSF | Uric acid, GABA | UA levels were low and GABA levels were high | Levels of Uric acid and GABA normalised post shunting |
| **Spanu [9]** | 1989 | NPH | 13 | NO | CSF | MHPG, HVA, 5-HIAA | Values were compared with those of obstructive hydrocephalus and di not differ | CSF levels of monoaminergic metabolites do not appear useful in the selection of patients for shunting. |
| **Yamada [10]** | 1991 | NPH (secondary) | 6 | YES | Serum | Melatonin | Preoperative levels were lower than controls. Diurnal rhythm was lost. | Shunting restored the diurnal rhythm of melatonin |
| **Molins [11]** | 1991 | NPH (and other dementias) | 5 | YES | CSF (lumbar) | Somatostatin | Levels of somatostatin were decreased in the NPH group | The decreased levels might indicate neuronal destruction or damage. |
| **Wikkelso [12]** | 1991 | NPH (and other dementias) | 10 | YES | CSF (lumbar) | Delta-sleep-inducing peptide (DSIP), vasoactive intestinal peptide (VIP), peptide YY (PYY) and somatostatin (SOM) | The concentration of DSIP, PPY and SOM were significantly lower than controls. DSIP, VIP and SOM increased significantly in parallel to the clinical improvement after the shunt operation in NPH patients. | Cognitive improvement was not correlated with the levels of these peptides in the CSF. Previous correlation of cognitive improvement with VIP levels was not reproduced successfully. |
| **Malm [13]** | 1991 | NPH (and other dementias) | 15 | YES | CSF | Cholinester-ase, Monoamine metabolites, lactate | Levels of monoamines did not differ between NPH, AD, MID and control | Low CSF outflow conductance may facilitate the clearance of these substances. |
| **Hildebrand [14]** | 1992 | NPH | 24 | NO | CSF (ventricular) | Homovanillic acid |  | Values were not correlated to any of the clinical features |
| [**Longatti**](http://www.ncbi.nlm.nih.gov/entrez/query.fcgi?db=pubmed&cmd=Search&term="Longatti+PL"%5BAuthor%5D) **[15]** | 1993 | Adult | 17 | NO | CSF (ventricular) | Myelin Basic Protein (MBP) | Raised values of ventricular CSF concentration of MBP demonstrated a significant decrease after shunt operation | Important index of actual brain damage in hydrocephalus and could be taken in account for the indication of shunt operation. |
| **Catalan [16]** | 1994 | NPH | 7 | YES | CSF (lumbar) | Neuropeptide Y | Lower levels in NPH when compared to controls. No correlation with MMSE score | NPY is not specific for NPH, as it is also lower in patients with AD |
| [**Nooijen**](http://www.ncbi.nlm.nih.gov/entrez/query.fcgi?db=pubmed&cmd=Search&term="Nooijen+PT"%5BAuthor%5D) **[17]** | 1997 | NPH (and other dementias) | 43 | YES | CSF | Neuron-specific enolase (NSE), S-100, Myelin basic protein (MBP) and lactate | 57 hydrocephalic patients were assessed. The levels of the proteins did not differ significantly between patients and controls | A single determination of CSF concentration of these brain-specific proteins was of little value in the differential diagnosis of the dementia syndrome. In the diagnosis of normal pressure hydrocephalus increased levels of CSF lactate may be helpful for the differential diagnosis with other dementias. |
| **Galard [18]** | 1997 | Adult | 16 | YES | CSF (lumbar) | Cholecysto-kinin (CCK) | Reduced levels of CCK when compared with controls. Abnormal ICP values were correlated with low levels of CCK | ICP alterations are responsible for the loss of CCK in adult hydrocephalus |
| **Tullberg [19]** | 1998 | NPH | 65 | YES | CSF (lumbar) | Neurofilament triplet protein (NFL), Glial fibrillary acidic protein (GFAP) | NFL levels ↑ six-fold and GFAL levels ↑ twofold when com pared with controls. NFL levels were associated with favourable outcome post shunting. | NFL is a marker of ongoing axonal damage in NPH. |
| **Tullberg [20]** | 2000 | NPH (and SAE) | 43 | NO | CSF (lumbar) | Sulfatide, NFL, GFAP, VIP, 4-aminobutyric acid (GABA), Homovanillic acid (HVA),  5-hydroxy-indoleacetic acid (5-HIAA),  4-hydroxy-3-methoxyphenylglycol  (HMPG)) | The patients with NPH with cerebrovascular aetiology had higher sulfatide concentrations and a poorer outcome after shunt surgery than patients with NPH with other aetiologies | The CSF sulfatide concentration distinguished between patients with SAE and those with NPH with a sensitivity of 74% and a specificity of 94%, making it an important diagnostic marker |
| **Kudo [21]** | 2000 | NPH | 20 | YES | CSF (ventricular) | Tau protein | ↑ Tau levels compared to controls | Tau levels were correlated with severity of dementia, urinary incontinence and gait disturbance. May determine the level of neuronal degeneration. |
| **Poca [22]** | 2001 | NPH | 14 | NO | CSF (lumbar) | Neuropeptide Y (NPY), somatostatin  (SOM), and corticotropin releasing factor  (CRF) | SOM and CRF  concentrations were significantly increased  in all patients post shunting. Concentrations of  NPY were increased in 12/14 patients. | Shunting can restore SOM,  NPY, and CRF concentrations even in  patients with longstanding normal pressure  hydrocephalus. The difference in biochemistry did not correlate with ambulation and daily  life activities, and cognitive performance. |
| **Mase [23]** | 2003 | NPH (and other dementias) | 14 | YES | CSF (lumbar) | Lipocalin-type Prostaglandin D synthase (PGDS) | Lower levels in NPH than controls and patients with other dementias. | Useful marker for the differential diagnosis o NPH from other disorders with dementia |
| **Tarkowski [24]** | 2003 | NPH | 35 | YES | CSF (lumbar) | TNF-α, sulfatide, neurofilament | Preoperative levels of TNF-α were increased when compared to controls. Shunting lead to complete disappearance of intrathecal TNF- α | TNF-α levels correlate with clinical improvement following shunting. |
| **Mataro [25]** | 2003 | NPH (idiopathic) | 8 | NO | CSF (lumbar) | Galanin | CSF galanin concentrations decreased post shunting | The postoperative decrease correlated with improvement in clinical status and cognitive functioning. |
| **Tisell [26]** | 2004 | AS, NPH | 19 | NO | CSF (ventricular) | Neurofila-ment light protein, tau protein, sulfatide, vasoactive intestinal peptide, neuropeptides PYY, CSF albumin | No difference in any of the markers levels were found between AS and INPH. This suggests similarities in pathophysiology | High albumin ratio and sulfatide concentrations in vCSF have negative implications for surgical outcome |
| **Fersten [27]** | 2004 | NPH | 24 | YES | CSF (lumbar) | Thiobarbituric acid-reactive material, protein sulfhydryl groups | TBAR, total & soluble protein groups levels in NPH higher than controls. Decreased levels of SH in NPH | Results indicate processes that activate the peroxidation of free radicals in NPH. |
| **Lins [28]** | 2004 | NPH | 12 | YES | CSF (lumbar) | Amyloid beta peptide ((1-42)) (Abeta42-IR) and total tau protein (TTIR) | TTIR in NPH was not significantly changed compared with VD, PD and controls. NPH-Abeta42-IR was significantly decreased compared with PD and controls | Combined measurement of Abeta42-IR and TTIR contributes to the differential diagnosis of NPH vs. Alzheimer’s Disease. |
| **Brettschneider [29]** | 2004 | NPH (and other dementias) | 19 | YES | CSF (lumbar) | Leptomeningeal derived beta trace protein, beta2 microglobulin and Cystatin C | Patients with NPH showed significantly lower b-trace protein levels than normal controls and Alzheimer’s patients. No difference in microglobulin and cystatin C levels between different groups | Leptomeningeal dysfunction may be involved in the pathogenesis of NPH |
| **Stoeck [30]** | 2005 | NPH (and other dementias) | 5 | YES | CSF (source not mentioned) | IL-4 and IL-10 | Higher levels when compared to controls | Elevated levels reflect either a response to neurodegeneration, or to neuroregeneration. |

**References for Table 1**

[1] Hammer M, Sorensen PS, Gjerris F, Larsen K. Vasopressin in the cerebrospinal fluid of patients with normal pressure hydrocephalus and benign intracranial hypertension. Acta Endocrinol (Copenh). 1982 Jun;100(2):211-5.

[2] Sutton LN, Wood JH, Brooks BR, Barrer SJ, Kline M, Cohen SR. Cerebrospinal fluid myelin basic protein in hydrocephalus. J Neurosurg. 1983 Sep;59(3):467-70.

[3] Sorensen PS, Vilhardt H, Gjerris F, Warberg J. Impermeability of the blood-cerebrospinal fluid barrier to 1-deamino-8-D-arginine-vasopressin (DDAVP) in patients with acquired, communicating hydrocephalus. Eur J Clin Invest. 1984 Dec;14(6):435-9.

[4] Sorensen PS, Gjerris F, Ibsen S, Bock E. Low cerebrospinal fluid concentration of brain-specific protein D2 in patients with normal pressure hydrocephalus. J Neurol Sci. 1983 Dec;62(1-3):59-65.

[5] Ahlberg J, Blomstrand C, Ronquist G, Wikkelso C. Dementia--and adenylate kinase activity in cerebrospinal fluid. Acta Neurol Scand. 1985 Nov;72(5):525-7.

[6] Albrechtsen M, Sorensen PS, Gjerris F, Bock E. High cerebrospinal fluid concentration of glial fibrillary acidic protein (GFAP) in patients with normal pressure hydrocephalus. J Neurol Sci. 1985 Oct;70(3):269-74.

[7] Wikkelso C, Fahrenkrug J, Blomstrand C, Johansson BB. Dementia of different etiologies: vasoactive intestinal polypeptide in CSF. Neurology. 1985 Apr;35(4):592-5.

[8] Kanki T. [Biochemical investigation of normal pressure hydrocephalus in the assessment of shunt effectiveness]. No To Shinkei. 1988 Feb;40(2):163-70.

[9] Spanu G, Santagostino G, Marzatico F, Gaetani P, Silvani V, Rodriguez y Baena R. Idiopathic hydrocephalic dementia in aging brain the neurosurgical approach. Funct Neurol. 1989 Jul-Sep;4(3):293-8.

[10] Yamada N, Iwasa H, Mori S, Kurokawa N, Fujimoto K, Kawashima K, et al. Melatonin secretion in normal pressure hydrocephalus after cerebral aneurysm rupture--investigation before and after ventriculoperitoneal shunt. Neurol Med Chir (Tokyo). 1991 Aug;31(8):490-7.

[11] Molins A, Catalan R, Sahuquillo J, Castellanos JM, Codina A, Galard R. Somatostatin cerebrospinal fluid levels in dementia. J Neurol. 1991 Jun;238(3):168-70.

[12] Wikkelso C, Ekman R, Westergren I, Johansson B. Neuropeptides in cerebrospinal fluid in normal-pressure hydrocephalus and dementia. Eur Neurol. 1991;31(2):88-93.

[13] Malm J, Kristensen B, Ekstedt J, Adolfsson R, Wester P. CSF monoamine metabolites, cholinesterases and lactate in the adult hydrocephalus syndrome (normal pressure hydrocephalus) related to CSF hydrodynamic parameters. J Neurol Neurosurg Psychiatry. 1991 Mar;54(3):252-9.

[14] Hildebrand J, Moussa Z, Raftopoulos C, Vanhouche J, Laute MA, Przedborski S. Variations of homovanillic acid levels in ventricular cerebrospinal fluid. Acta Neurol Scand. 1992 May;85(5):340-2.

[15] Longatti PL, Canova G, Guida F, Carniato A, Moro M, Carteri A. The CSF myelin basic protein: a reliable marker of actual cerebral damage in hydrocephalus. J Neurosurg Sci. 1993 Jun;37(2):87-90.

[16] Catalan R, Sahuquillo J, Poca MA, Molins A, Castellanos JM, Galard R. Neuropeptide Y cerebrospinal fluid levels in patients with normal pressure hydrocephalus syndrome. Biol Psychiatry. 1994 Jul 1;36(1):61-3.

[17] Nooijen PT, Schoonderwaldt HC, Wevers RA, Hommes OR, Lamers KJ. Neuron-specific enolase, S-100 protein, myelin basic protein and lactate in CSF in dementia. Dement Geriatr Cogn Disord. 1997 May-Jun;8(3):169-73.

[18] Galard R, Poca MA, Catalan R, Tintore M, Castellanos JM, Sahuquillo J. Decreased cholecystokinin levels in cerebrospinal fluid of patients with adult chronic hydrocephalus syndrome. Biol Psychiatry. 1997 Apr 1;41(7):804-9.

[19] Tullberg M, Rosengren L, Blomsterwall E, Karlsson JE, Wikkelso C. CSF neurofilament and glial fibrillary acidic protein in normal pressure hydrocephalus. Neurology. 1998 Apr;50(4):1122-7.

[20] Tullberg M, Mansson JE, Fredman P, Lekman A, Blennow K, Ekman R, et al. CSF sulfatide distinguishes between normal pressure hydrocephalus and subcortical arteriosclerotic encephalopathy. J Neurol Neurosurg Psychiatry. 2000 Jul;69(1):74-81.

[21] Kudo T, Mima T, Hashimoto R, Nakao K, Morihara T, Tanimukai H, et al. Tau protein is a potential biological marker for normal pressure hydrocephalus. Psychiatry Clin Neurosci. 2000 Apr;54(2):199-202.

[22] Poca MA, Mataro M, Sahuquillo J, Catalan R, Ibanez J, Galard R. Shunt related changes in somatostatin, neuropeptide Y, and corticotropin releasing factor concentrations in patients with normal pressure hydrocephalus. J Neurol Neurosurg Psychiatry. 2001 Mar;70(3):298-304.

[23] Mase M, Yamada K, Shimazu N, Seiki K, Oda H, Nakau H, et al. Lipocalin-type prostaglandin D synthase (beta-trace) in cerebrospinal fluid: a useful marker for the diagnosis of normal pressure hydrocephalus. Neurosci Res. 2003 Dec;47(4):455-9.

[24] Tarkowski E, Tullberg M, Fredman P, Wikkelso C. Normal pressure hydrocephalus triggers intrathecal production of TNF-alpha. Neurobiol Aging. 2003 Sep;24(5):707-14.

[25] Mataro M, Poca MA, Del Mar Matarin M, Catalan R, Sahuquillo J, Galard R. CSF galanin and cognition after shunt surgery in normal pressure hydrocephalus. J Neurol Neurosurg Psychiatry. 2003 Sep;74(9):1272-7.

[26] Tisell M, Tullberg M, Mansson JE, Fredman P, Blennow K, Wikkelso C. Differences in cerebrospinal fluid dynamics do not affect the levels of biochemical markers in ventricular CSF from patients with aqueductal stenosis and idiopathic normal pressure hydrocephalus. Eur J Neurol. 2004 Jan;11(1):17-23.

[27] Fersten E, Gordon-Krajcer W, Glowacki M, Mroziak B, Jurkiewicz J, Czernicki Z. Cerebrospinal fluid free-radical peroxidation products and cognitive functioning patterns differentiate varieties of normal pressure hydrocephalus. Folia Neuropathol. 2004;42(3):133-40.

[28] Lins H, Wichart I, Bancher C, Wallesch CW, Jellinger KA, Rosler N. Immunoreactivities of amyloid beta peptide((1-42)) and total tau protein in lumbar cerebrospinal fluid of patients with normal pressure hydrocephalus. J Neural Transm. 2004 Mar;111(3):273-80.

[29] Brettschneider J, Riepe MW, Petereit HF, Ludolph AC, Tumani H. Meningeal derived cerebrospinal fluid proteins in different forms of dementia: is a meningopathy involved in normal pressure hydrocephalus? J Neurol Neurosurg Psychiatry. 2004 Nov;75(11):1614-6.

[30] Stoeck K, Bodemer M, Ciesielczyk B, Meissner B, Bartl M, Heinemann U, et al. Interleukin 4 and interleukin 10 levels are elevated in the cerebrospinal fluid of patients with Creutzfeldt-Jakob disease. Arch Neurol. 2005 Oct;62(10):1591-4.
